# Mitochondrial Sirt3 serves as a biomarker for sepsis diagnosis and mortality prediction

**DOI:** 10.1038/s41598-022-14365-w

**Published:** 2022-06-21

**Authors:** Jingjing Liu, Gaosheng Zhou, Rongping Chen, Zewen Tong, Hongmin Zhang, Xiaoting Wang, Dawei Liu

**Affiliations:** Department of Critical Care Medicine, Peking Union Medical College Hospital, Peking Union Medical College, Chinese Academy of Medical Sciences, 1# Shuai Fu Yuan, Dong Cheng District, Beijing, 100730 China

**Keywords:** Diagnostic markers, Predictive markers, Prognostic markers

## Abstract

The purpose of this study is to determine whether the levels of serum Sirt3 correlate with disease severity and perfusion indicators in septic patients, as well as to assess the clinical value of Sirt3 as a potential novel marker for sepsis diagnosis and mortality prediction. A total of 79 patients in the ICU were included in the study, of which 28 were postoperatively noninfectious and the remaining 51 patients were all diagnosed with sepsis during the study period. The levels of Sirt3 were detected and dynamically monitored by enzyme-linked adsorption method, Pearson or Spearman coefficient for correlation analysis between Sirt3 and clinical indicators, ROC curve for evaluation of diagnosis and mortality prediction, Kaplan–Meier method for the significance of Sirt3 in 28-day survival. The serum levels of Sirt3 were lower in the sepsis patients on day 1 (P < 0.0001), and the septic shock group had lower Sirt3 levels than the sepsis group (P = 0.013). Sirt3 had good negative correlations with SOFA scores both in sepsis and septic shock groups (Pearson: r^2^ = − 0.424, − 0.518; P = 0.011, 0.040), and Sirt3 correlated strongly with ScvO_2_ in the septic shock group (Pearson: r^2^ = − 0.679, P = 0.004) and with PCT in the sepsis group (Pearson: r^2^ = − 0.409, P = 0.015). Sirt3 not only performed well in identifying sepsis (AUC = 0.995, 95% CI 0.987–1, P < 0.0001) but also greatly enhanced lactate's specificity in detecting septic shock (from 91.43 to 94.29%). Patients in the low Sirt3 group had higher ScvO_2_, lactate, APACHE II score, SOFA score, longer ICU stays, and worse indicators of inflammation (TNF-α, IL-6) and infection (PCT) than those in the high Sirt3 group (P < 0.05). Additionally, Sirt3 can predict mortality of sepsis (AUC = 0.746, 95% CI 0.571–0.921, P = 0.022), patients with serum Sirt3 < 10.07 pg/ml have a lower 28-day survival (log-rank P = 0.008). Low serum levels of Sirt3 are significantly correlated with the disease severity. At the same time, Sirt3 increases the sensitivity of lactate to detect “cellular hypoxia” in septic shock. Sirt3 is a promising biomarker for the diagnosis of sepsis and predicting mortality risk in septic patients.

## Introduction

Sepsis is a multi-organ failure that is caused by the body's dysregulated response to infection, and elevated serum lactate levels and circulatory failure constitute the core of septic shock^1^. Lactate levels, as well as their trends over time, are reliable indicators of the severity of sickness and mortality^2,3^. We now recognize that cytokine signaling^4^, mitochondria damage^5^, catecholamine stimulation^6,7^, and other factors^8^ can cause the rising of lactate in septic shock, and tissue hypoxia is no longer the main explanation of elevated lactate. Researchers have demonstrated that oxygen delivery to tissue is sufficient during sepsis, but increases in circulating lactate are still common in septic patients due to the limited ability of tissues to utilize oxygen. This hypoxia phenomenon caused by the inability of cells to utilize oxygen is called “Cytopathic hypoxia”^9,10^, which highlights the critical role of mitochondrial function in raising lactate levels during sepsis^11^.

Several pathways of mitochondria damage can lead to “Cytopathic hypoxia”. These involve damaging mitochondria by ROS from mitochondria themselves^12^, changing mitochondrial protein expression^13,14^, suppression of key enzymes in the tricarboxylic acid cycle (TCA)^15^, inhibition of mitochondrial enzyme complexes^16–18^, and exhaustion of the pyruvate dehydrogenase complex (PDC) activity^19,20^. In sepsis, mitochondrial dysfunction contributes to adverse outcomes, such as multiple organ dysfunction syndromes (MODS)^21^ and even death^22^.

Sirt3 is a NAD-dependent deacetylase found in mitochondrial-rich organs such as the kidney, heart, and brain^23^. Sirt3 can control the activity of metabolic enzymes such as succinate dehydrogenase (SDH)^24^, long-chain acyl-coenzyme A dehydrogenase (LCAD)^25^ to regulate the TCA^26^, oxidative phosphorylation^27^, and fatty acid metabolism pathways^28^ in mitochondria^28^. Sirt3 also plays a vital role in controlling the pattern of mitochondria function within cells, as well as the cell's adaptive response to metabolic stress^29^. The dysregulation of Sirt3 is also implicated in diseases involving mitochondria, such as cancer^30^, cardiac disease^29,31^, various metabolic disorders^31^, and aging^32,33^. Some studies have reported the clinical value of SIRT3 expression in various types of cancer^34,35^.

Similarly, Sirt3 also has been found in sepsis to protect various organs through its regulation of mitochondrial function, including inhibiting vascular inflammation^36^, protecting the pulmonary endothelial barrier function^37^, preventing cardiac insufficiency^38,39^, and also reducing renal^40^ and intestinal injury^41^. Sirt3 also contributes to regulating the immune metabolic switch between hyper-inflammatory and hypo-inflammatory during sepsis^42^. However, Sirt3 has been rarely studied in patients with sepsis. Based on the above background, we speculated that Sirt3 may be a useful marker for the recognition of “Cytopathic hypoxia” during sepsis. Therefore, in this prospective, non-interventional cohort study, we first described the levels of Sirt3 in the serum of patients with sepsis or septic shock, explored the correlation between serum Sirt3 levels and severity of illness, clinical perfusion indicators, and infection indicators, as well as the importance of Sirt3 as an indicator of diagnosing and predicting in septic patients.

## Method

### Design

The prospective observational clinical study was conducted at Peking Union Medical College Hospital from May 2021 to December 2021 which was approved by the PUMCH institutional review board (approval number JS-3302). We obtained informed consent from all enrolled patients through the next of kin of each patient. Our study was performed in accordance with the Declaration of Helsinki and was guided by the sepsis 3.0 diagnostic criteria and Clinical Nursing Practice Guidelines (2018) to ensure the safety of human subjects and sample compliance. We screened and categorized 79 patients within 24 h after their admission to the intensive care unit (ICU) based on strict inclusion and exclusion criteria, including 35 patients with sepsis, 16 patients with septic shock, and 28 patients without infection.

### Patients

All patients in the study were older than 18 years. Patients with sepsis must meet the latest diagnostic criteria (Sepsis-3 defines sepsis as “life-threatening organ dysfunction caused by a dysregulated host response to infection; Sequential [Sepsis-related] Organ Failure Assessment Score (SOFA) is used to define organ dysfunction as an increase in the total SOFA score of 2 points or more)^10^. Patients with sepsis will be classified into the septic shock group when they meet the following criteria: persisting hypotension requiring vasopressors to maintain mean arterial pressure [MAP] > 65 mmHg and having serum lactate level > 2 mmol/L despite adequate volume resuscitation. Patients in the ICU control group need to have normal infection indicators.

The enrolled septic patients were assessed on each study day to determine if they remained in sepsis or septic shock. During the study, patients who were transferred from ICU due to abandonment of treatment or improvement of their condition, as well as those who were no longer diagnosed with sepsis in the ICU, were not studied on subsequent study days (D3, D5, or D7).

Patients who had been or were currently diagnosed with cancer, including all types of tumors, were excluded from the study. Patients who are pregnant were also excluded.

### Serum collection

We collected arterial blood through an arterial indwelling catheter. Septic patients were collected on days 1, 3, 5, and 7 after being admitted to the ICU, while patients in the control group were only collected on day 1. Blood collected in the anticoagulant tube was centrifuged at 3000 r/min for 10 min after standing for 10 min, then transferred to an EP tube and stored at − 80 °C.

### Clinical data collection

The patient's admission information (age, gender, primary disease, history, infection site, Length of hospital stay, Length of ICU stay), basic vital signs (blood pressure, heart rate, respiratory rate, body temperature), biochemical examination (white blood cells, hemoglobin, PCT, IL-6, IL-10, hs-CRP), SOFA, APACHE II, ventilator parameters, antibiotics, vasoactive drugs, lactate, central venous oxygen saturation (ScvO_2_), and other information were also collected.

### Elisa for serum Sirt3 level

After defrosting the frozen serum, we used the human Sirt3 ELISA kit (Cusabio Biotech Co, Ltd, Wuhan, China, Catalog # CSB-EL021341HU) to detect the concentration of Sirt3 in the serum.

### Statistical analysis

We used SPSS 26.0 and GraphPad PRISM 8.0 for statistical analysis and plotting. The Shapiro–Wilk test tested whether the data were normally distributed. The normally distributed data were expressed as mean ± standard, and compared by the One-way Anova or T-test. Non-normal distribution data were expressed as M (P25, P75), and compared by the Kruskal–Wallis test or Mann–Whitney U test. And a variance homogeneity test was also performed, and non-parametric tests were used when variances are uneven. The counting data were expressed as rates (%), and the comparison between groups was tested by χ^2^. A Pearson correlation coefficient analysis was for normally distributed data, a Spearman rank correlation coefficient analysis was for non-normally distributed data. ROC curves were used to analyze the diagnostic efficacy of various indicators. 28-day survival was estimated by the Kaplan–Meier method, and differences in survival were evaluated with the log-rank test. P < 0.05 means that the difference between the two groups is statistically significant.

## Results

### Baseline characteristics

A total of 79 patients were included in this study, 51 of whom were diagnosed with sepsis during the study period, and 28 were non-infected patients postoperatively. We obtained baseline data on three groups of patients (Table 1) after grouping by diagnosis on day 1. There were 35 patients in the sepsis group (57.88 ± 20.78 years, 60% male), 16 in the septic shock group (64.06 ± 17.02, 75% male), and 28 in the ICU control group (58.25 ± 20.70, 60.7% male). The past medical history was counted in all groups (sepsis VS septic shock VS ICU control), including hypertension (31.4% vs. 56.25% vs. 46.43%), diabetes mellitus (22.9% vs. 18.75% vs. 25%), hyperlipidemia (11.4% vs. 6.25% vs. 17.86%), heart attack (11.4% vs. 18.75% vs. 39.29%), cerebral hemorrhage (5.7% vs. 6.25% vs. 10.71%). The sites of infection of patients in the sepsis group and septic shock group were also counted: lung (20% vs. 6.25%), abdomen (34.29% vs. 50%), central nervous system (11.42% vs. 12.5%) bloodstream infection (14.29% vs. 12.5%), skin (5.7% vs 6.25%) or others (14.29% vs 12.5%). According to the follow-up of 28-day mortality, 9 septic patients died with a mortality rate of 17.65%, including 4 in the sepsis group (11.42%) and 5 in the septic shock group (37.5%).

**Table 1:** Baseline characteristics of all patients on day 1.

	Sepsis (n = 35)	Septic shock (n = 16)	ICU control (n = 28)	P value
Sex [male] (%)	21 (60%)	12 (75%)	17 (60.7%)	
Age (years)	57.88 ± 20.78	64.06 ± 17.02	58.25 ± 20.70	0.587
MAP (mmHg)	81.58 ± 10.94	84.38 ± 6.84	86.36 ± 10.36	0.173
HR (bpm)	89.09 ± 17.836	100.44 ± 14.02	78.18 ± 15.38	< 0.05
CVP (cmH_2_O)	6.91 ± 2.17	9.12 ± 2.19	–	< 0.05
**past medical history (n, %)**				
Hypertension	11 (31.4%)	9 (56.25%)	13 (46.23%)	
Diabetes	8 (22.9%)	3 (18.75%)	7 (25%)	
Hyperlipidemia	4 (11.4%)	1 (6.25%)	5 (17.86%)	
Cardiovascular disease	4 (11.4%)	3 (18.75%)	11 (39.29%)	
Cerebral infarction	2 (5.7%)	1 (6.25%)	3 (10.71%)	
**Site of infection (n, %)**				
Pneumonia	7 (20%)	1 (6.25%)	–	
Peritonitis	12 (34.29%)	8 (50%)	–	
Central system infection	4 (11.42%)	2 (12.5%)	–	
Bloodstream infection	5 (14.29%)	2 (12.25%)	–	
Skin infections	2 (5.7%)	1 (6.25%)	–	
Others	5 (14.29%)	2 (12.25%)	–	
SOFA score	9.37 ± 3.34	13.35 ± 2.828	1.57 ± 2.659	< 0.05
APACHE II score	18 ± 7.68	21.5 ± 7.95	13.5 ± 6.46	< 0.05
NE (ug/kg/min)	0.049 (0,0.568)	0.798 (0.071,1.15)	–	< 0.05
Lactate (mmol/L)	1.2 (0.6,2.6)	3.5 (2.1,9.7)	0.6 (0.8,3)	< 0.05
P_(V-A)_CO_2_ (mmHg)	2.9 (1.65,4.6)	3.2 (1.25,4.88)		0.828
ScvO_2_ (%)	74.17 ± 8.14	75.87 ± 10.03	–	0.54
OI	314.58 ± 125.46	268.41 ± 118.83	374.70 ± 132.47	< 0.05
PCT (ng/ml)	15.34 ± 23.45	16.97 ± 23.65	0.67 ± 1.29	< 0.05
WBC (10^9^/L)	14.24 ± 7.68	10.68 ± 9.07	11.78 ± 2.55	0.155
TNF-α (mg/L)	17.49 ± 12.84	30.80 ± 15.71	–	< 0.05
IL-10 (pg/ml)	7.35 (5.175,109.5)	15.6 (7.24,52.3)	–	0.108
IL-6 (pg/ml)	136.49 ± 267.47	446.41 ± 87.45	–	< 0.05
CTNI (ng/ml)	5.32 ± 21.38	40.64 ± 151.21	0.18 ± 0.02	0.402
NT-proBNP (pg/ml)	6075.47 ± 9225.54	7972.45 ± 10,137.53	893.85 ± 700.20	0.244
Length of ICU stay (days)	8 (3.5,8.75)	12 (8,43)	1 (1,17)	< 0.05
Length of hospital stay (days)	27 (5,121)	34.5 (3,88)	9 (3,38)	< 0.05
28-day mortality rate (n,%)	4 (11.42%)	5 (37.5%)	0	< 0.05

Values are expressed as mean ± standard deviation, or median (interquartile range), or number (percentage). P < 0.05 were considered statistically significant.

*MAP* mean arterial pressure, *HR* heart rate, *CVP* Central venous pressure, *SOFA Score* Sequential Organ Failure Assessment Score, *APACHE II score* Acute Physiology and Chronic Health Evaluation II score, *NE* Norepinephrine, *P*_*(V-A)*_*CO*_*2*_ central venous-to-arterial carbon dioxide difference, *ScvO*_*2*_ Central venous oxygen saturation, *OI* oxygenation index, *PCT* procalcitonin, *WBC* white blood cell, *TNF-α* Tumor Necrosis Factor α, *IL-10* Interleukin-10, *IL-6* Interleukin-6, *CTNI* cardiac troponin I, *NT-proBNP* N-terminal (NT)-prohormone Brain natriuretic peptide.

During the assessment of the patients on the first day, vital signs (heart rate, blood pressure), infection indicators (WBC, PCT), inflammatory indicators (TNF-α, IL-10, IL-6), CVP, Norepinephrine (NE), oxygenation index (OI), CTNI, and NT-pro BNP were collected. Except for MAP, P_(V-A)_CO_2_, ScvO_2_, WBC, IL-10, CTNI, and NT-pro BNP, the rest of the indicators were statistically significant (Table 1).

### Characteristics of changes in serum levels of Sirt3 in patients with ICU

**Figure 1:**
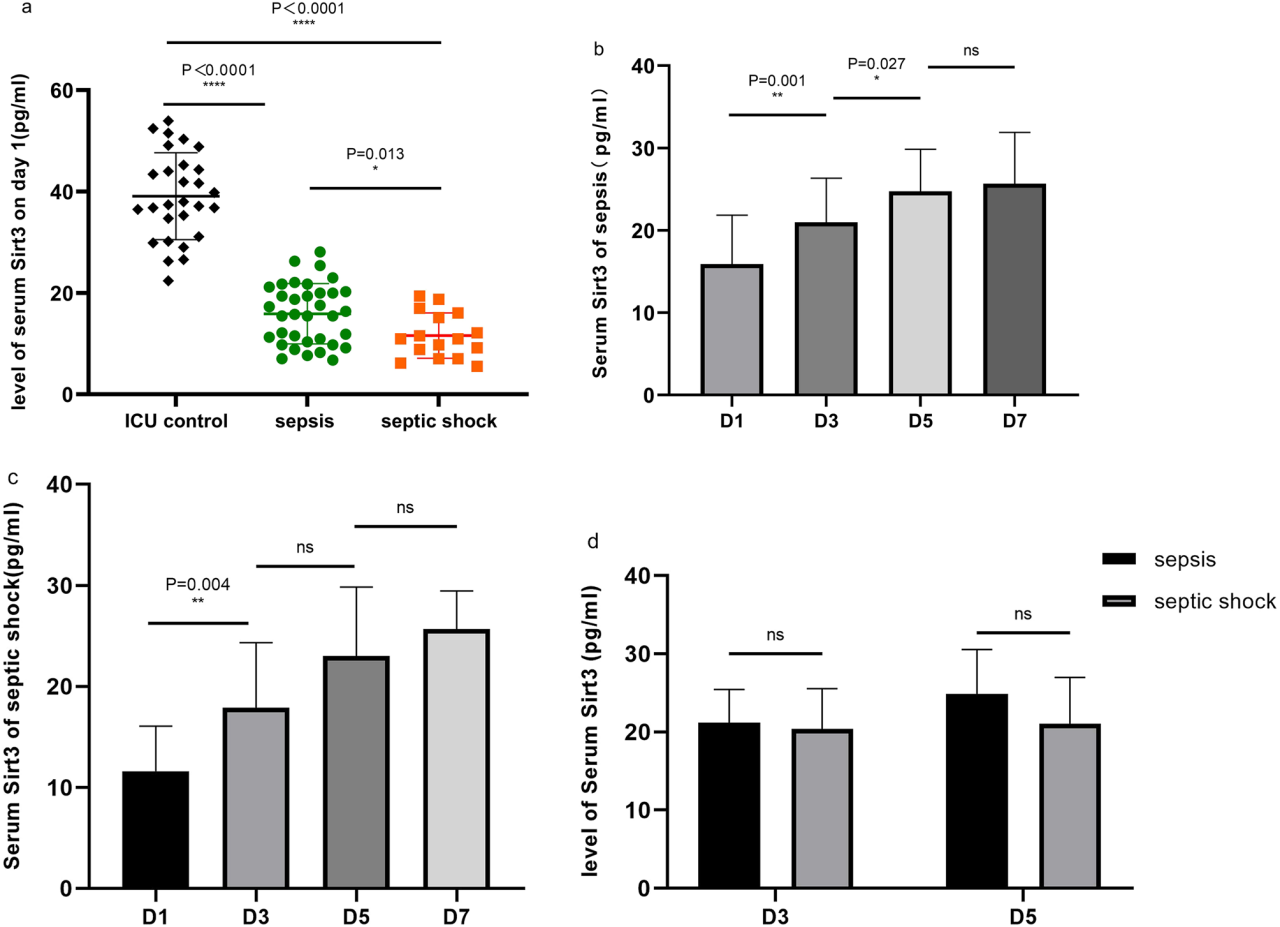
Characteristics of changes in serum levels of Sirt3 in patients with ICU. (**a**) Serum Sirt3 levels were measured in sepsis group, septic shock group, and ICU control group. (**b**) Serum Sirt3 levels on day1, day3, day5, and day7 in patients diagnosed with sepsis on day 1. (**c**) Serum Sirt3 levels on day1, day3, day5, and day7 in patients diagnosed with septic shock on day 1. (**d**) Serum Sirt3 levels were measured in sepsis group and septic shock group after re-diagnosed on D3 and D5. P < 0.05 were considered statistically significant. *denotes P < 0.05, **denotes P < 0.01, ****denotes P < 0.0001, “ns” means no significance.

The serum level of Sirt3 was lower in septic patients on day 1 (P < 0.0001), and the septic shock group had a lower level than the sepsis group (P = 0.013) (Fig. 1a).

We tracked changes in Sirt3 serum levels on days 3, 5, and 7. The Sirt3 levels in the sepsis group gradually increased over time, and there were statistical differences in growth between D3 and D1 (P = 0.001), D5 and D3 (P = 0.027), but there was no difference between D5 and D7 (P = 0.671) (Fig. 1b). A gradual increase in Sirt3 levels over time was also observed in the septic shock group, where there was a statistical difference between D3 and D1 (P = 0.004), but no statistical difference between D5 and D3, D7 and D5 (P = 0.052, 0.324) (Fig. 1c).

We re-diagnosed and re-grouped the patients on D3, D5, and D7 to confirm that the diagnosis was as accurate as feasible because the patients' conditions may interchange after therapy. On D3, 10 patients were transferred from the ICU, and 41 patients were studied, including 30 patients with sepsis (25 patients with sepsis and 5 patients with septic shock at D1), 11 patients with septic shock (2 patients with sepsis and 9 patients with septic shock at D1). On the fifth day, 11 patients left the ICU, and the remaining 30 patients included 23 patients with sepsis (18 patients with sepsis and 5 patients with septic shock at D3) and 7 patients with septic shock (2 patients with sepsis and 5 patients with septic shock at D3). On D7, 11 patients were moved from the ICU and 19 patients remained, including 17 patients with sepsis (14 with sepsis and 3 with septic shock from D5) and 2 with septic shock (from the septic shock group at D5). The difference in Sirt3 in the two groups was then compared again on days 3 and 5, but there was no statistical difference (Fig. 1d). Since there were only 2 patients in the septic shock group on D7 after being re-diagnosed, the difference between the two groups had a low confidence level and was therefore not shown.

### Correlation between Sirt3 and clinical indicators in ICU patients

Our analysis found significant differences in Sirt3 levels among the three groups of patients on day 1, so we analyzed the correlation between Sirt3 levels and clinical indicators among the three groups on day 1.

**Figure 2:**
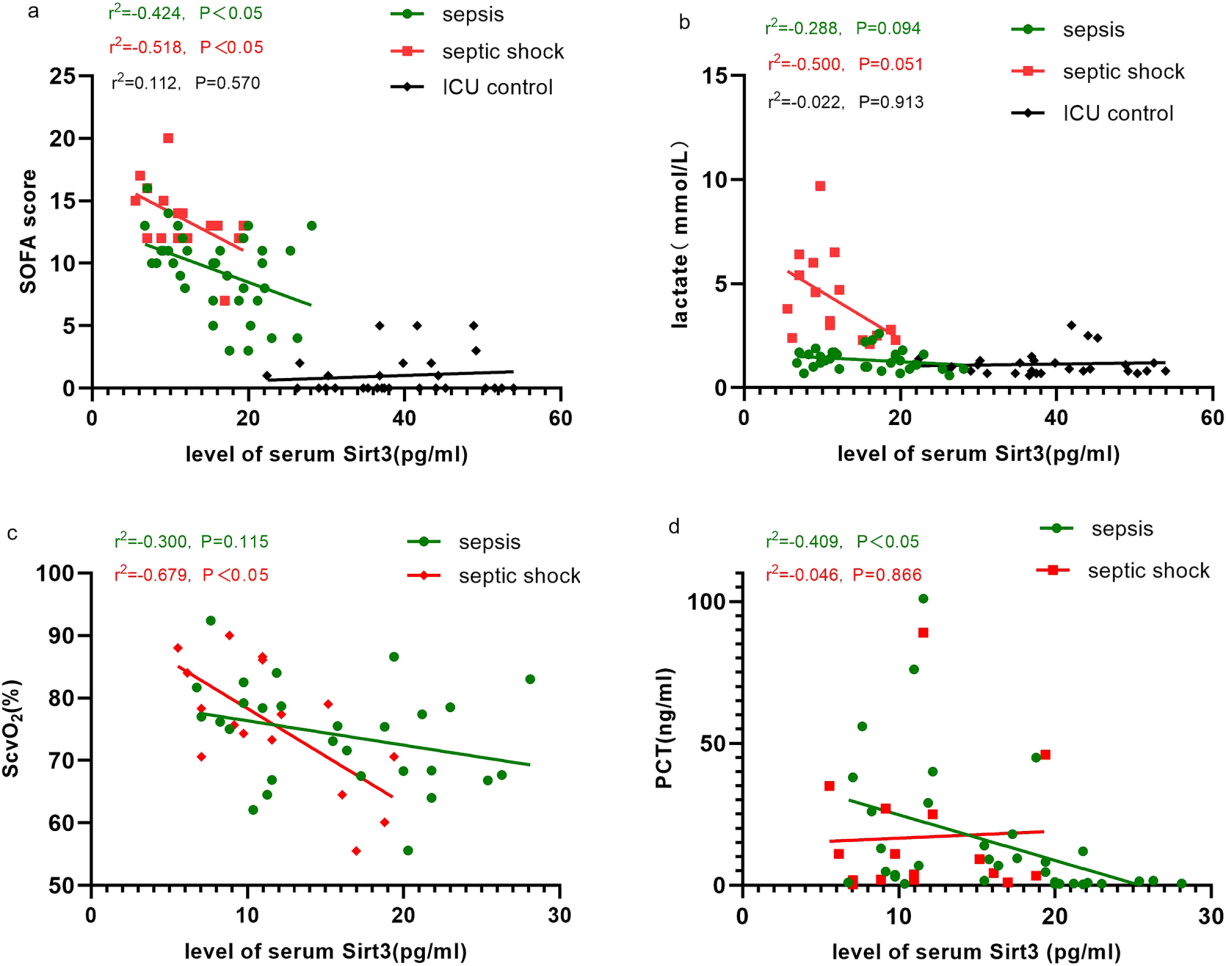
Correlation between Sirt3 and clinical indicators in ICU patients. (**a**) Correlation between serum Sirt3 and SOFA score in sepsis group, septic shock group, and ICU control group. (**b**) Correlation between serum Sirt3 and lactate in sepsis group, septic shock group, and ICU control group. (**c**) Correlation between serum Sirt3 and ScvO_2_ in sepsis group and septic shock group. (**d**) Correlation between serum Sirt3 and PCT in sepsis group and septic shock group. P < 0.05 were considered statistically significant. *SOFA* Sequential Organ Failure Assessment Score, *ScvO*_*2*_ Central venous oxygen saturation, *PCT* procalcitonin.

Our first analysis was to determine if Sirt3 correlated with organ dysfunction, and we found that Sirt3 had a negative correlation with SOFA scores both in sepsis and septic shock groups (Pearson: r^2^ = − 0.424, − 0.518; P = 0.011, 0.040), but no significant correlation with the ICU controls (Pearson: r^2^ = 0.112, P = 0.570) (Fig. 2a). No correlation was found between Sirt3 and APACHE II scores in any of the three groups (Pearson: r^2^ = − 0.381, − 0.064, − 0.075 P = 0.143, 0.713, 0.705).

The correlation analysis between Sirt3 and clinical indicators of perfusion revealed that Sirt3 did not correlate with Lactate in the three groups (Spearman: r^2^ = − 0.500, − 0.288, 0.022 P = 0.051, 0.094, 0.913) (Fig. 2b). However, Sirt3 correlated well with ScvO2 in the septic shock group of patients (Pearson: r^2^ = − 0.679, P = 0.004), but had no correlation with the sepsis group (Pearson: r^2^ = − 0.300, P = 0.115) (Fig. 2c). Similarly, Sirt3 did not correlate with P_(V-A)_CO_2_ in either group (Spearman: r^2^ = − 0.084, − 0.197; P = 0.664, 0.46). Data for ScvO_2_ and P_(V-A)_CO_2_ were not obtained because central venous access was not established in patients in the ICU control group.

The correlation between serum Sirt3 and PCT was examined in the groups with sepsis (Pearson: r^2^ = − 0.409, P = 0.015), but not in the groups with septic shock (r^2^ = 0.046, P = 0.866) (Fig. 2d).

### The potential value of Sirt3 in the diagnosis of sepsis and septic shock

**Figure 3:**
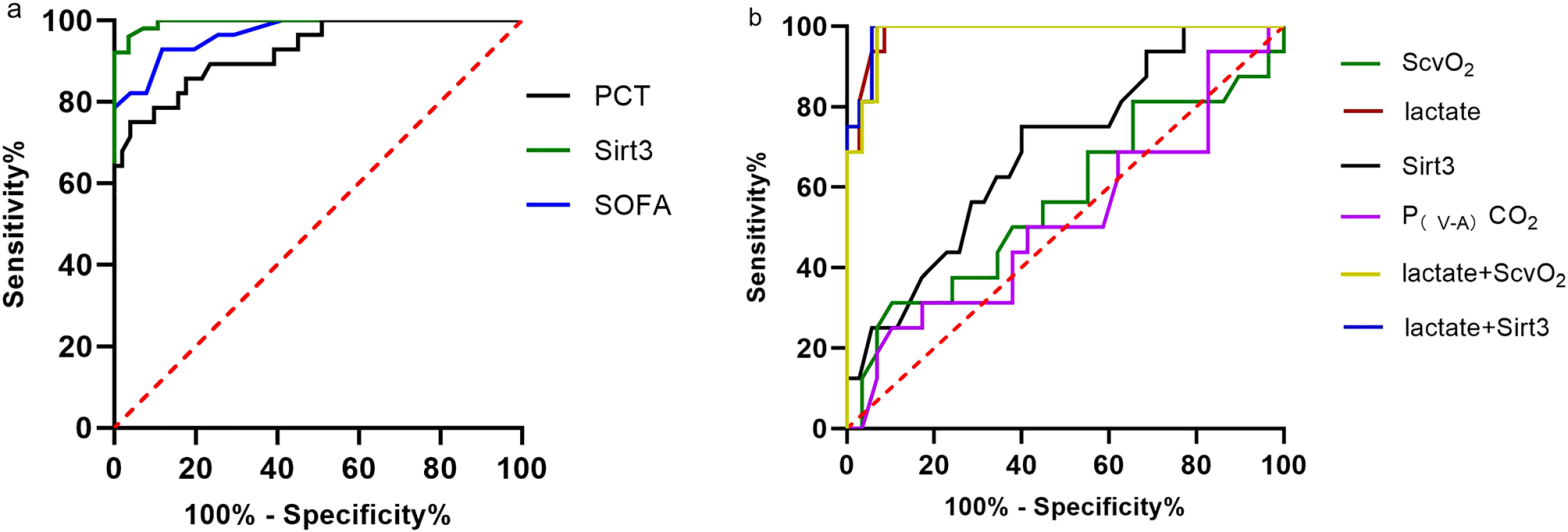
The ROC curves of indicators for the diagnosis of sepsis and the early detection of sepsis from septic shock. (**a**) The ROC curves of indicators for the diagnosis of sepsis (PCT, Sirt3, SOFA). (**b**) The ROC curves of indicators for the early detection of septic shock (lactate, Scvo_2_, Sirt3, lactate + Sirt3, Lactate + ScvO_2_, P_(V-A)_CO_2_). Calculate the predicted values for lactate + Sirt3 and lactate + ScvO2 by binary logistic regression and then calculate the ROC curve. *SOFA* Sequential Organ Failure Assessment Score, *ScvO*_*2*_ Central venous oxygen saturation, *PCT* procalcitonin, *P*_*(V-A)*_*CO*_*2*_ central venous-to-arterial blood carbon dioxide partial pressure.

The ROC curves of Sirt3 for the diagnosis of sepsis and septic shock were also displayed. Sirt3 performed better in identifying sepsis (AUC = 0.995, 95% CI 0.987–1, P < 0.0001) than SOFA (AUC = 0.968, 95% CI 0.934–0.1, P < 0.0001), PCT (AUC = 0.925, 95% CI 0.865–0.985, P < 0.0001) (Fig. 3a). And based on the cutoff value of 25.85 pg/ml Sirt3 yielded sensitivity and specificity were 96.08% and 96.43%.

In screening septic shock patients from septic patients, we discovered that Sirt3 (AUC = 0.69, 95% CI 0.537 − 0.843, P = 0.031) has better performance than ScvO_2_ and P_(V-A)_CO_2_ (Fig. 3b, Table2), and lactate's specificity in detecting septic shock could be greatly enhanced (from 91.43% to 94.29%) when lactate was utilized in conjunction with Sirt3 (Fig. 3b, Table2).

**Table 2:** The AUC and optimal study parameter cutoff points for different indicators of septic shock and their associated diagnostic and efficacy values.

	AUC ± SE	P-value	Cut-off value	Sensitivity (%)	Specificity (%)
Lactate	0.986 ± 0.012	< 0.001	2 (mmol/l)	100%	91.43%
ScvO_2_	0.567 ± 0.094	0.46	83.5 (%)	31.25%	89.66%
Sirt3	0.690 ± 0.078	< 0.05	15.33 (pg/ml)	75%	60%
P_(V-A)_CO_2_	0.518 ± 0.094	0.84	1.25 (mmHg)	25%	89.66%
Lactate + Sirt3	0.988 ± 0.011	< 0.001	–	100%	94.29%
Lactate + ScvO_2_	0.982 ± 0.015	< 0.001	–	100%	93.1%

*ScvO*_*2*_ Central venous oxygen saturation, *P*_*(V-A)*_*CO*_*2*_ central venous-to-arterial carbon dioxide difference. *AUC* the area under the ROC curve; *Cut-off value* the optimal cutoff points for the sample diagnosis of septic shock. P < 0.05 means statistically significant.

### Difference of clinical indicators in different serum levels of Sirt3

We got a cutoff value (15.33 pg/ml) from the maximal Youden index of the ROC curve for screening septic shock. We divided the patients into low Sirt3 (< 15.33 pg/ml) and high Sirt3 (≥ 15.33 pg/ml) groups, with 25 patients in the low Sirt3 group (13 sepsis and 12 septic shock) and 26 patients in the high Sirt3 group (22 sepsis and 4 septic shock). After comparing and assessing the differences in clinical indicators between the two groups, we discovered that patients in the low Sirt3 group had higher APACHE II and SOFA scores, longer ICU stays, worse indicators of inflammation (TNF-α, IL-6) and infection (PCT) than those in the high Sirt3 group (Table3).

**Table 3:** Comparative clinical data grouped by cutoff value (15.33 pg/ml) in patients with sepsis.

	Low Sirt3 (n = 25)	High Sirt3 (n = 26)	P value
HR (bpm)	94.8 ± 17.98	90.57 ± 16.97	0.392
MAP (mmHg)	84.04 ± 10.53	80.92 ± 9.11	0.263
CVP (cmH_2_O)	7.48 ± 2.16	7.84 ± 2.57	0.585
OI	305.18 ± 109.72	295.21 ± 138.59	0.778
HB (g/l)	99.68 ± 31.21	98.85 ± 22.83	0.913
RASS	− 1.96 ± 1.43	− 2.58 ± 1.55	0.372
NE (ug/kg/min)	0.23 (0.056,0.49)	0.096 (0,0.231)	0.203
PEEP (ml/cmH_2_O)	5 (5,6.75)	5 (4.5,8)	0.873
**Diagnosis (n,%)**			
Sepsis	13 (52%)	22 (84.6%)	
Septic shock	12 (48%)	4 (15.4%)	
**Severity of illness**			
SOFA score	12.84 ± 2.68	8.69 ± 3.27	< 0.001***
APACHE II score	21.2 ± 7.97	16.8 ± 7.00	0.045*
Length of ICU stay (days)	8 (5,10)	14 (8,29)	0.035*
Length of hospital stay (days)	26 (14.5,40.75)	27.5 (14,42.25)	0.713
**Perfusion indicators**			
P_(V-A)_CO_2_ (mmHg)	2.5 (1.625,4.775)	3.5 (1.45,4.6)	
Lactate (mmol/l)	1.9 (1.5,4.6)	1.25 (1,2.025)	0.003*
ScvO_2_ (%)	79.16 ± 6.86	69.78 ± 8.14	< 0.001***
**Laboratory parameters of infection**			
PCT ( (ng/ml))	68 (13,87)	2.45 (0.675,9.4)	0.019*
Hs-CRP (mg/l)	190 (188.41,190)	190 (190,190)	0.865
WBC (10^9^)	11.37 ± 7.52	13.62 (7.8,17.05)	0.137
**Inflammatory cytokines**			
TNF-α (mg/l)	24.56 (18.35,34.15)	24.37 (12.87,24.55)	0.033*
IL-10 (pg/ml)	75.53 (15.6,86.763)	35 (6.15,86.76)	0.144
IL-8 (pg/ml)	278.5 (85.25,366)	197 (74.75,366)	0.315
IL-6 (pg/ml)	209.6 (108,244)	137.5 (39.73,209.6)	0.024*

Values are expressed as mean ± standard deviation, median (interquartile range), or number (percentage).*denotes P < 0.05, ***denotes P < 0.001

*MAP* mean arterial pressure, *HR* heart rate, *CVP* Central venous pressure, *OI* oxygenation index, *SOFA Score* Sequential Organ Failure Assessment Score, *APACHE II score* Acute Physiology and Chronic Health Evaluation II score, *P*_*(V-A)*_*CO*_*2*_ central venous-to-arterial carbon dioxide difference, *HB* Hemoglobin, *NE* Norepinephrine, *RASS* Richmond Agitation-Sedation Scale, *P*_*(CV-A)*_*CO*_*2*_ central venous-to-arterial blood carbon dioxide partial pressure, *ScvO2* Central venous oxygen saturation, *OI* oxygenation index, *PCT* procalcitonin, *Hs-CRP* high-sensitivity C-reactive protein, *WBC* white blood cell, *TNF-α* Tumor Necrosis Factor α, *IL-10* Interleukin-10, *IL-8* Interleukin-8, *IL-6* Interleukin-6.

Mostly, It’s interesting that even though parameters impacting ScvO_2_ and lactate (RASS score, hemoglobin, OI, heart rate, P_(V-A)_CO_2_, blood pressure, CVP, NE) did not differ, ScvO_2_ and lactate showed significant difference between the two groups (p < 0.001) (Table3).

### Predictive value of Sirt3 for 28-day survival

**Figure 4:**
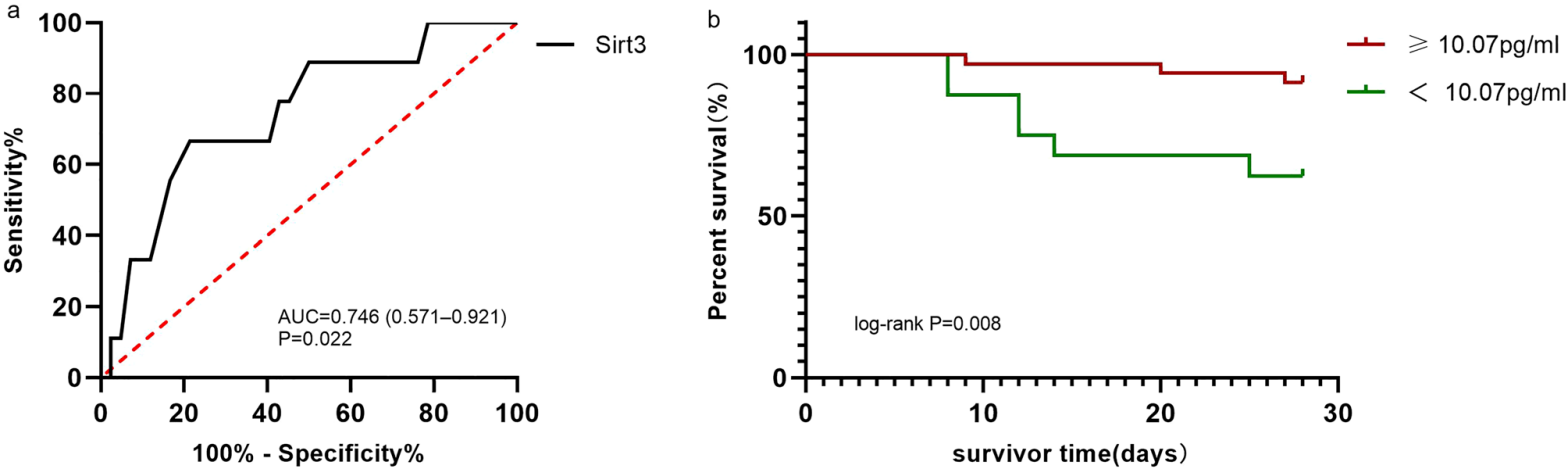
The ROC curves of predicting for 28-day survival and Kaplan–Meier estimator analysis of Sirt3 for 28 day survival. (**a**) The ROC curves of Sirt3 for the predicting 28-day mortality in patients with sepsis. P < 0.05 were considered statistically significant (**b**) Kaplan–Meier estimator analysis of Sirt3 for 28-day survival. The cut off value of Sirt3 for the estimated 28-day mortality of patients with sepsis was 10.07 pg/ml.

A total of 9 patients died during the 28-day follow-up period in our research, we plotted the ROC curve for Sirt3 predicted mortality. The area under the curve for Sirt3 was 0.746 (95% CI 0.571–0.921, P = 0.022, Fig. 4a). Based on the cutoff value of 10.07 pg/ml of Sirt3 yielded sensitivity and specificity were 66.67% and 78.57%, and all the patients were divided into two groups.

Kaplan–Meier estimator analysis showed the 28-day survival rate of patients with low Sirt3 was markedly reduced than that of patients with high Sirt3 (log-rank P = 0.008, Fig. 4b).

## Discussion

In our research, Sirt3 showed good clinical significance in sepsis. Sirt3 correlated with PCT in the sepsis group and was substantially linked with Scvo2 in the septic shock group, and its levels were negatively correlated with illness severity. Sirt3 also improved the specificity of lactate in the diagnosis of septic shock. It was an acceptable diagnostic marker for identifying sepsis and predicting mortality risk in septic patients. Furthermore, patients with low levels of Sirt3 had a worse prognosis and lower 28-day survival.

Sirt3 is a member of the Sirtuins family, which is composed of seven highly conserved NAD-dependent deacetylases that could regulate cell survival^43^, metabolism^44^, and longevity^45^. Our research showed a strong correlation between Sirt3 and SOFA, which is consistent with the results of most basic experiments on sepsis-induced MODS. In the study of septic cardiomyopathy, Sirt3 can activate AMPK-related mitochondrial biogenesis^39^ and acetylate important enzymes in the tricarboxylic acid cycle^29^ to reduce sepsis-induced myocardial injury in mice. Sirt3-AMPK signaling is also important for suppressing vascular inflammation and endothelial dysfunction during early sepsis^36^. Lack of Sirt3 increases CLP-induced renal insufficiency, tubular cell death and apoptosis, mitochondrial dysfunction, and ROS production in a knockout mouse model of AKI^46^. These studies focused on the cellular energy metabolism pathway have helped us understand Sirt3's role in protecting organ function at the cellular level, and our research demonstrated a close correlation between the serum level of Sirt3 and MODS in sepsis patients. MODS has been suggested to be caused by mitochondrial damage and energy metabolism disorders in cells during sepsis^47^, thus producing a phenomenon similar to “hibernation”^48^, which closely joins energy metabolism to MODS. Sirt3 contains the switches that control mitochondrial energy metabolism, it may be a potential strategy for future sepsis treatments that target mitochondria.

As one of the diagnostic criteria for septic shock, lactate is used as a perfusion indicator^49^ and a resuscitation therapy endpoint^50^ in clinical practice, although it can also signify numerous causes of non-perfusion. No correlation between Sirt3 and lactate was found in our study, and the reasons for this result may include small sample size and a complex mechanism of lactate elevation. Lactate is a ubiquitously produced and utilized metabolite that has a core place in nearly all energy-related pathways in humans. There is no consensus on the source, clearance, and metabolic functions of lactate in sepsis, and even we are not certain if high levels of lactate provide protection in sepsis. Understanding the pathophysiology and clinical significance of lactate is difficult. Hyperoxia is no longer a complete explanation for elevated lactate in sepsis, and mitochondrial dysfunction and decreased PDC activity have also been questioned. Research indicated^51^ that the early elevation in muscle lactate concentration during LPS infusion was not attributable to the mitochondrial dysfunction or PDC inhibition. Even if the mitochondrial function is normal, lactate levels appear to rise when pyruvate generation surpasses PDC to Acetyl Coenzyme A conversion. Elevated lactate is more likely to be a metabolic stress response under stress rather than just the result of cellular oxygenation problems in sepsis. Faced with the complexity of lactate, Sirt3, an indicator of regulating mitochondrial metabolism, is difficult to achieve desirable results in studies with small sample sizes. We still hope to have a large sample study in the future to investigate the correlation between Sirt3 and lactate.

Even though the cause of lactate production is not sufficiently clear, mitochondrial dysfunction remains a significant element in the formation of increased lactate during the septic shock phase. Lactate responds too slowly to be used to guide acute changes in sepsis^52^, Sirt3 may be an earlier indicator of the phenomenon of cellular hypoxia than lactate. So we tried to explore the potential of Sirt3 for identifying patients at risk of developing septic shock whose lactate cannot be distinguished from sepsis timely, but unfortunately, due to only 2 sepsis patients progressed to septic shock when we regrouped (on D3 and D5) during the study, we did not get such results. We may need to find a larger sample size to verify again. But we found that Sirt3 compensates for lactate deficiency in response to the phenomenon of *"*cytopathic hypoxia*"* in septic shock. Sirt3 allows high levels of circulating lactate as an indicator of impaired cellular oxygen metabolism.

ScvO_2_ is an indicator of the balance between oxygen supply and oxygen consumption, and it can be easily disturbed by several factors, such as mitochondria, hemoglobin, cardiac output, oxygen partial pressure, and patient agitation. But ScvO_2_ cannot provide a clear explanation for the imbalance of systemic oxygen supply and demand, which is the limitation of ScvO_2_, especially during septic shock. Current research suggests that impaired microcirculation and mitochondria function cause both an insufficient supply and ineffective utilization of oxygen, and these two factors affect tissue oxygen utilization independently^53^. We found that Sirt3 has a strong correlation with ScvO_2_, but this correlation remains limited to the septic shock group, which reflects poorer cellular oxygen utilization during septic shock. Mitochondria are the main site for the utilization of oxygen, we discovered that there was a significant difference in ScvO_2_ between the low Sirt3 and high Sirt3 groups when the factors that influence ScvO_2_ did not differ statistically, but this result did not appear between the sepsis group and septic shock groups, which implied that Sirt3 had the potential to be an indicator of cellular oxygen utilization in response to septic shock.

The massive release of inflammatory factors is closely related to mitochondrial metabolism. During sepsis, immune cells switch to glycolysis, numerous metabolites (like lactate, citrate, and succinate), produced by glycolysis and fractured TCA, accumulate in the cell to facilitate signaling and epigenetic interactions which drive the inflammatory response and secrete a large number of inflammatory factors (TNF-α, IL-6)^54^. Sirt3 is the bridge between mitochondrial metabolism and immune cell activity, and downregulation of Sirt3 reprograms mitochondrial metabolism and promotes macrophage death^55^. Sirt3 can also affect immune cell survival by regulating mitochondrial homeostasis^56^. Our results showed that the lower the level of Sirt3, the worse the indicators of inflammation (TNF-α, IL-6) and infection (PCT), a result that was expected.

Mitochondria's role in diseases is no longer limited to provide energy to cells, they also play a role in signaling, gene control, cellular calcium regulation, and activation of cell death pathways. Sirt3 influences mitochondrial intracellular function through the homeostatic regulation of mitochondrial fission^57^, biosynthesis^58^, and autophagy^59^, in addition to altering their metabolic activity. The results of this study predicted that patients with lower serum Sirt3 levels have a worse prognosis, and our survival analysis backed up this prediction. The clinical significance of Sirt3 in sepsis was confirmed by our study.

## Limitation

Despite the findings, there are still many limitations. In our study, we didn't find a stronger correlation between Sirt3 and the sepsis group, and we also didn't find a correlation between lactate and Sirt3, as well as we failed to use Sirt3 to identify patients at risk of developing septic shock from sepsis, which could be due to the limited sample size. This is a single-center study, so generalizability may be limited, and it's also unclear whether different treatments influenced the findings. In the future, we hope for bigger multicenter follow-up cohort studies to look into the role of Sirt3 in sepsis.

## Conclusion

Sirt3 is a promising biomarker for the diagnosis of sepsis and predicting mortality risk in septic patients. Low serum levels of Sirt3 are significantly correlated with the disease severity. In septic shock, Sirt3 can bridge the gap in the specific response of lactate to the *"*cytopathic hypoxia*"*. Patients with low levels of Sirt3 have a worse prognosis and lower 28-day survival.

## Data Availability

The datasets generated and analyzed during the current study are available from the corresponding author on reasonable request.
